# A quantitative assay to monitor HSV-1 ICP0 ubiquitin ligase activity *in vitro*

**DOI:** 10.1016/j.ymeth.2015.04.004

**Published:** 2015-11-15

**Authors:** Chris Boutell, David J. Davido

**Affiliations:** a MRC-University of Glasgow Centre for Virus Research, 464 Bearsden Road, Glasgow G61 1QH, UK; b Department of Molecular Biosciences, University of Kansas, Lawrence, KS 66049, USA

**Keywords:** HSV-1, ICP0, RING, Ubiquitin, Infrared, LI-COR

## Abstract

The ubiquitin–proteasome system is an essential cellular process that plays a fundamental role in the regulation of protein stability. This pathway is tightly controlled by a sequential cascade of enzymatic steps that culminates in the formation of a poly-ubiquitin chain onto the substrate protein targeted for 26S proteasome degradation. Through a process of co-evolution viruses have evolved mechanisms to utilize or suppress this pathway in order to enhance their replication and spread. One of the first proteins to be expressed during herpes simplex virus 1 (HSV-1) infection is ICP0, a viral RING-finger E3 ubiquitin ligase that targets a variety of cellular proteins for ubiquitination and proteasome-dependent degradation. This activity is required in order for ICP0 to efficiently stimulate the onset of HSV-1 lytic infection and viral reactivation from latency. While it is clear that the RING-finger domain of ICP0 plays an important role in the biology of HSV-1, methods for accurately quantifying its biochemical activity are currently lacking. Here we describe a protocol that enables the quantitative measurement of the ubiquitin ligase activity of ICP0 using near-infrared (IR) western blot imaging. The use of such imaging technology provides an accurate means to examine the biochemical and kinetic parameters of RING-finger ubiquitin ligases in solution, and may provide significant application for inhibitor studies.

## Highlights

• Application of near-infrared imaging to quantify ubiquitin biochemistry *in vitro*.
• A quantitative methodology to monitor E3 ubiquitin ligase activity in solution.
• Validation of sensitivity and dynamic linear range.
• Applicability to E3 ubiquitin ligase inhibitor studies.

## 1 Introduction

**Fig. 1:**
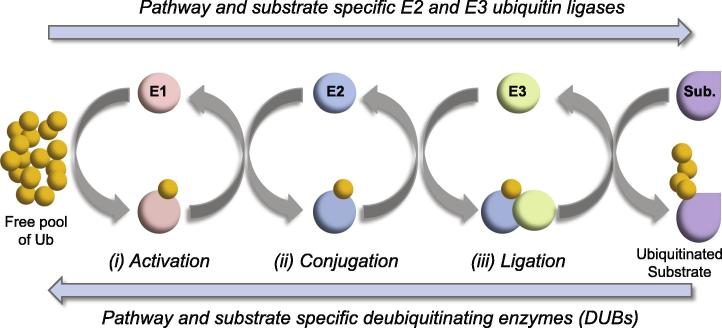
Schematic illustration highlighting the salient features of the ubiquitin pathway. The post-translational modification of proteins through the covalent attachment of ubiquitin (Ub) occurs through a sequential cascade of enzymatic events: (i) activation through ubiquitin E1 activating enzymes. (ii) Conjugation through pathway specific E2 ubiquitin conjugating enzymes. (iii) Ligation to substrate (Sub.) through pathway and substrate specific E3 ubiquitin ligases. Modification typically occurs through the formation of an isopeptide bond between ubiquitin and a solvent exposed lysine residue within the substrate. Illustration depicts the formation of a poly-ubiquitin chain through the sequential modification of the anchored ubiquitin molecule within the substrate. Ubiquitin modification is dynamic and reversible (blue arrows) through the activity of pathway specific deubiquitinating enzymes (DUBs).

The ubiquitin pathway is an essential cellular process in all eukaryotes. The post-translational modification (PTM) of proteins through the covalent attachment of ubiquitin, itself a small 76 amino acid protein, is known to play a fundamental role in the regulation of many aspects of cell biology. The process of ubiquitin modification (ubiquitination) requires an ATP-dependent cascade of enzymatic steps; including ubiquitin activation, conjugation, and ligation by E1, E2, and E3 pathway component enzymes, respectively (schematically summarized in Fig. 1). E3 ubiquitin ligases not only provide substrate-specificity, but also assist in the enzymatic transfer of ubiquitin from charged E2 ubiquitin conjugating enzymes onto substrate proteins undergoing modification [1]. The attachment of ubiquitin typically occurs through the formation of an isopeptide bond between the C-terminal glycine residue of ubiquitin and the amino side chain of a solvent exposed lysine residue within the substrate protein. This initial mono-ubiquitination event can be subsequently extended through the sequential addition of ubiquitin molecules onto lysine residues within the attached ubiquitin molecule itself, leading to the formation of an anchored poly-ubiquitin chain (Fig. 1; reviewed in [2]). As ubiquitin contains seven lysine residues, each of which can serve as an acceptor site for ubiquitin attachment, a structurally diverse range of ubiquitin chain types can be formed that influence various aspects of protein biochemistry and cell biology; including the cell cycle (Lysine-11 linked chains), proteasome-mediated degradation (Lysine-48 linked chains), as well as DNA repair and host immune signaling pathways (Lysine-63 linked chains; reviewed in [3]).

Due to the importance of the ubiquitin–proteasome system, viruses have evolved mechanisms to manipulate this pathway to their advantage in order to enhance their replication and spread (reviewed in [4], [5]). One of the first proteins to be expressed during herpes simplex virus 1 (HSV-1) infection is ICP0 (infected cell protein 0), a C3HC4 RING-finger E3 ubiquitin ligase [6]. Genetic studies have shown that ICP0 is required to efficiently stimulate the onset of HSV-1 lytic infection and productive reactivation of viral genomes from latency (reviewed in [7], [8]). These biological phenotypes are tightly linked to its biochemical properties as a viral E3 ubiquitin ligase and its ability to interact with host-cell E2 ubiquitin conjugating enzymes [9], [10], [11]. ICP0 mediates the ubiquitination and proteasome-dependent degradation of numerous cellular proteins through a variety of targeting mechanisms (reviewed in [7]). These activities inhibit a range of cellular processes and pathways; including intrinsic and innate host immunity [10], [12], [13], [14], [15], [16], [17], [18], [19], [20], [21], SUMO modification [12], [22], [23], centromere stability and the cell cycle [24], [25], [26], [27], [28], and aspects associated with DNA repair [29], [30], [31], [32], [33] amongst others. The ubiquitin ligase and substrate targeting properties of ICP0 have also been shown to be influenced by its phosphorylation status [34], [35], affecting the outcome of HSV-1 pathogenesis and reactivation from latency in animal model systems *in vivo* [36].

The RING-finger domain of ICP0 interacts with both class I (UBE2D1/UbcH5a) and class II (UBE2E1/UbcH6) E2 ubiquitin conjugating enzymes [6], [9], [37], and to efficiently catalyze the formation of poly-ubiquitin chains on a variety of different substrate proteins; including itself (auto-ubiquitination; [6], [38]), USP7 [39], p53 [40], RNF8 [31], [33], poly-SUMO2 chains [22], and PML [15], [41], both *in vitro* and *in vivo*. Traditionally, the biochemical properties of ICP0 as a RING-finger ubiquitin ligase have been monitored using standard electrochemiluminescence (ECL)-based western blot assays. However, quantitation of ICP0 biochemical activity using this approach is limited due to poor detection efficiencies of ubiquitinated products and the restricted linear range of chemiluminescent signal with traditional western blot approaches. Consequently, we have established a sensitive and quantitative protocol to examine the kinetics of ICP0 biochemistry *in vitro* through the use of near-infrared (IR) imaging of immunoblots probed with fluorescent secondary antibodies. This method can be used to quantitatively assess the biochemical properties of RING-finger ubiquitin ligases in solution, their respective phenotypes following mutation, and inhibitor studies thereof. This methodology will therefore be useful in the identification and characterization of specific regions within ICP0 that influence its biochemical activity and its corresponding ability to stimulate the onset of HSV-1 lytic infection and viral reactivation from latency.

## 2 Material and methods

### 2.1 Recombinant proteins

Polyhistidine-tagged UBE2D1 (UbcH5a) and Glutathione-*S*-transferase (GST)-tagged ICP0.241 (amino acids 1–241 of ICP0 encompassing the C3HC4 RING-finger domain) were purified from bacterial extracts using affinity isolation chromatography, as described previously [6]. Polyhistidine-tagged E1 ubiquitin-activating enzyme was purified from baculovirus-infected cell extracts, as described previously [6]. Wild-type ubiquitin (Sigma–Aldrich; U6253) and methylated-ubiquitin (BostonBiochem; U-501) were purchased from commercial sources.

### 2.2 *In vitro* ubiquitination assays

Ubiquitination assays were carried out in a final reaction volume of 10 μl in 50 mM Tris (pH 7.5), 50 mM NaCl, 1 mM MgCl_2_, and 5 mM ATP (Sigma–Aldrich; A7699) supplemented with 10 ng E1, 40 ng of E2 (UBE2D1), and 90 ng of E3 (GST-ICP0.241) per reaction. Reaction mixtures were activated by the addition of 1 μg of wild-type or methylated-ubiquitin per reaction and incubated at 37 °C for the specified times. For inhibition assays, reaction mixtures were incubated in the presence or absence of disodium dihydrogen ethylenediaminetetraacetic acid (EDTA) for 5 min prior to the addition of ubiquitin. Assays were terminated by the addition of 3× SDS–PAGE loading buffer supplemented with 8 M urea and 100 mM Dithiothreitol (DTT). Samples were heat denatured at 95 °C for 10 min prior to SDS–PAGE (12% Bis–Tris NuPAGE; Life Technologies). Proteins were transferred to 0.2 μm nitrocellulose membranes (GE Healthcare Life Sciences) using an XCell II transfer module (Life Technologies) for 60 min at 30 V.

### 2.3 Western blot assay

Membranes were blocked in 0.45 μm filtered phosphate buffered saline (PBS) supplemented with 10% fetal calf serum (FCS) for 60 min at room temperature. Primary and secondary antibody incubations were performed in filtered PBST–FCS (PBS supplemented with 0.1% Tween-20 and 10% FCS) at the desired antibody dilution (as stated below) for 1 h at room temperature. Membranes were sequentially washed three times in PBST for 5 min following each antibody incubation and three times in 0.2 μm filtered H_2_O prior to scanning. Primary antibodies: monoclonal anti-ICP0 (11060 [1/1000]; [42]), anti-ubiquitin (P4D1 [1/1000]; SantaCruz Biotech), and polyclonal anti-ICP0 (3678 [1/1000]; a kind gift from David Davido, University of Kansas). Secondary antibodies: goat anti-mouse and anti-rabbit IgG Dylight 800 and 680 (Cat# SA5–35521/35568 [1/20,000]; Thermo Scientific).

### 2.4 Quantitation and analyses of mono- and poly-ubiquitin products

Membranes were scanned using an Odyssey CLx infrared imaging system (LI-COR Biosciences) at a resolution of 84 μm and 0.0 mm focus offset. Bands or regions of interest (as highlighted) were quantified using Image Studio software (LI-COR) on raw unprocessed data files. Regression analysis, *R*-squared (*R*^2^), and 1/2 *V*_max_ values were calculated using Sigmaplot (Systat Software, Inc). Images were exported as 600 ppi RGB or grayscale TIF files, minimally processed in Adobe Photoshop, and annotated in Adobe Illustrator (Adobe Systems Incorporated).

## 3 Results

**Fig. 2:**
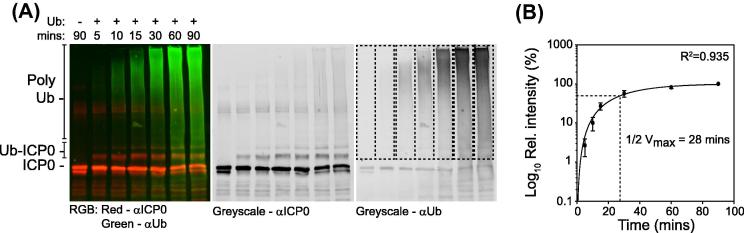
Quantitation of unanchored poly-ubiquitin chain formation by ICP0 using near-IR imaging. *In vitro* reaction mixtures containing E1, E2 (UBE2D1), and E3 (GST-ICP0.241) were activated through the addition of wild-type ubiquitin (±Ub) and incubated at 37 °C for the specified time (mins). Reaction mixtures were resolved by SDS–PAGE and analyzed by multiplex western blotting in conjunction with near-IR imaging for the formation of unanchored poly-ubiquitin chains (mAb P4D1 and Dylight anti-mouse 800; green) and ICP0 auto-ubiquitination (pAb 3678 and Dylight anti-rabbit 680; red). (A) Representative image of a scanned immunoblot showing RGB (left-hand panel) and corresponding single channel grayscale images (anti-ICP0 and anti-ubiquitin; middle and right-hand panels, respectively). Unmodified ICP0, auto-ubiquitinated ICP0 (Ub-ICP0), and unanchored poly-ubiquitin chains (Poly Ub) are highlighted. (B) Regions of interest (ROI; dashed boxes right-hand panel in A) relating to unanchored poly-ubiquitin chain formation were quantified and normalized with respect to the 90-min time point in the presence of ubiquitin within individual experiments. Scatter plot depicts the mean intensity for each time point. Bars represent the standard errors of the means (SEMs) from three independent experiments. Regression analysis, *R*^2^, and ½ *V*_max_ values were calculated using Sigmaplot (Systat Software, Inc).

One of the reported advantages of near-IR imaging over standard chemiluminescence based western blotting methodologies is the increased stability and dynamic range of its signal. In order to examine the application of such imaging technology to study the biochemical properties of ICP0 in solution, we performed a series of *in vitro* assays monitoring the ability of ICP0 to catalyze the formation of unanchored poly-ubiquitin chains and mono-ubiquitinated products in the presence of recombinant human UBA1 (E1) and UBE2D1 (E2) enzymes under established assay conditions [9]. Preliminary experiments demonstrated that secondary antibody optimization was required in order to reduce uneven fluorescent background signal across the membrane, which indirectly influenced the accurate quantitation of specific bands or regions of interest (data not shown). It is recommended, therefore, that both primary and secondary antibody concentrations are fully optimized prior to quantitation for consistent and reproducible results. Following a short series of optimization experiments, however, multiplex western blot assays analyzing the biochemical properties of ICP0 in solution were readily achievable. Time course experiments quickly established the advantages of near-IR imaging over standard film-based chemiluminescent methods by providing simultaneous outputs for both ICP0 auto-ubiquitination (Fig. 2A; red signal) and unanchored poly-ubiquitin chain formation (Fig. 2A; green signal). Quantification of three independent rounds of unanchored poly-ubiquitin chain formation demonstrated a strong correlation (*R*^2^ = 0.935) in the ability of ICP0 to stimulate the formation of unanchored poly-ubiquitin chains over time (1/2 *V*_max_ = 28 min), which spanned two orders of magnitude in relative intensity (Fig. 2B). Poly-ubiquitin chains were readily quantifiable within 5 min of assay activation even though their relative signal by eye was comparatively weak to that of ICP0 auto-ubiquitination (Fig. 2A and B).

**Fig. 3:**
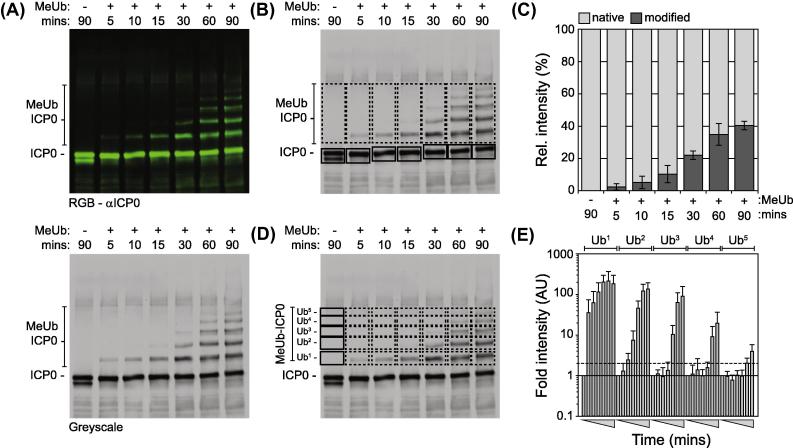
Quantitation of ICP0 auto-ubiquitination activity using near-IR imaging. Equivalent reaction mixtures (as described in Fig. 2) were activated by the addition of methylated-ubiquitin (±MeUb: a ubiquitin derivative unable to support poly-ubiquitin chain formation) and incubated for the specific times (mins) at 37 °C. Reaction mixtures were analyzed by western blotting for ICP0 auto-ubiquitination (MeUb ICP0; mAb 11060 and Dylight anti-mouse 800). (A) Representative image of a scanned immunoblot showing RGB and corresponding single channel grayscale image (top and bottom panels, respectively). (B) ROI relating to unmodified ICP0 (native; solid boxes) and total auto-ubiquitinated ICP0 (modified; dashed boxes) were quantified for their respective signal intensities and normalized with respect to equivalent areas of the membrane in the negative control (90-min time point in the absence of ubiquitin) within individual experiments. Bar graph depicts the relative intensity of native (light gray bars) to modified (dark gray bars) ICP0 as a proportion of the total signal intensity (%). Means and standard deviations in modified ICP0 signal intensity from three independent experiments are shown. (C) ROI relating to single lysine mono-ubiquitination events within ICP0 (dashed boxes; Ub^1^–Ub^5^) were individually quantified and normalized with respect to equivalent areas of the membrane in the negative control (90 min in the absence MeUb; solid boxes). Bar graph depicts the relative fold increase in individual lysine mono-ubiquitination within ICP0 over the time course of analysis (gray triangles). Black line depicts baseline following background normalization. Gray dotted line represents one standard deviation from background. Means and standard deviations from three independent experiments are shown. Images shown are representative and taken from a single experiment.

In order to examine the use of near-IR imaging in the quantification of substrate ubiquitination, biochemical assays were conducted in the presence of methylated-ubiquitin (MeUb): a derivative of ubiquitin unable to form poly-ubiquitin chains due to lysine methylation. As with wild-type ubiquitin (Fig. 2A), ICP0 was observed to readily undergo auto-ubiquitination in the presence of MeUb within 5 min of assay activation (Fig. 3A). Quantitation of ICP0 auto-ubiquitination over time demonstrated that 40% of the total input ICP0 was ubiquitinated within 90 min (Fig. 3B and C). Moreover, using near-IR imaging it was also possible to quantify the accumulation of individual auto-ubiquitination events over time (Fig. 3D and E). These data suggest that individual lysine residues within ICP0 may undergo auto-ubiquitination in a sequential manner, although future mutagenesis studies would be required to validate this hypothesis. Taken together, we conclude that near-IR imaging provides a sensitive and robust system to quantitatively analyze the ubiquitin ligase properties of ICP0 in solution.

**Fig. 4:**
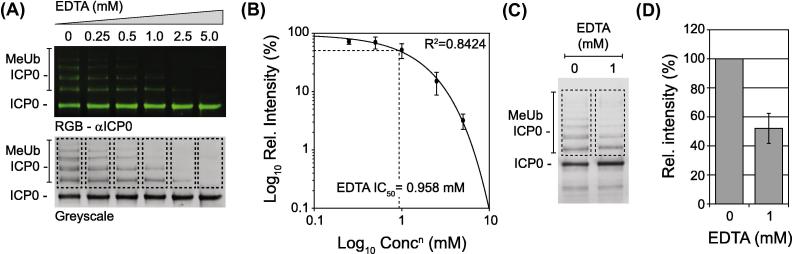
Quantification of ICP0 ubiquitin ligase activity inhibition using near-IR imaging. Equivalent reaction mixtures (as described in Fig. 3) were incubated in the presence of increasing concentrations of EDTA for five minutes prior to MeUb activation and incubation for 60 min at 37 °C. Samples were analyzed by western blotting for ICP0 auto-ubiquitination (MeUb ICP0; mAb 11060 and Dylight anti-mouse 800). (A) Representative image of a scanned immunoblot showing RGB and corresponding grayscale image (top and bottom panels, respectively). (B) ROI representing ICP0 auto-ubiquitination (dashed boxes in A) were quantified for their respective signal intensities and normalized within individual experiments with respect to the no drug control. Scatter plot depicts the mean intensity from three independent experiments at each concentration of EDTA (mM). Regression analysis, *R*^2^, and IC_50_ values were calculated using Sigmaplot (Systat Software, Inc). Bars represent SEMs from three independent experiments. (C and D) Validation of EDTA IC_50_ value. Equivalent reactions were performed in the presence or absence of 1 mM EDTA. Image highlights ROI used to quantify the levels of ICP0 auto-ubiquitination. Bar graph depicts the relative fold decrease in total ICP0 mono-ubiquitination in the presence of EDTA. Means and standard deviations from three independent experiments are shown.

Using standard chemiluminescence methods we have previously shown that EDTA (a non-specific divalent cation chelator) is able to competitively inhibit the RING-finger ubiquitin ligase activity of ICP0 in a dose-dependent manner *in vitro* [43]. In order to test the application of near-IR imaging for inhibitor screening, we performed an inhibitor study using EDTA. Reaction mixtures were incubated in the presence of increasing concentrations of EDTA for 5 min prior to assay activation and incubation in the presence of MeUb for 60 min (Fig. 4A). ICP0 auto-ubiquitination activity was quantified over three independent experiments and a dose–response curve calculated (Fig. 4B). Under these assay conditions, the 50% inhibitory concentration (IC_50_) for EDTA was calculated to be 0.958 mM. In order to validate this inhibitory dose, single concentration inhibitor assays were conducted and the levels of ICP0 auto-ubiquitination quantified relative to the no drug control (Fig. 4C). In the presence of 1 mM EDTA a 48% mean reduction in total levels of ICP0 auto-ubiquitination was observed (Fig. 4D), independently verifying the sensitivity and reproducibility of this assay system. We conclude that near-IR imaging provides a robust platform of technology to assess the efficacy of small molecule compounds to inhibit ICP0 RING-finger ubiquitin ligase activity *in vitro*.

## 4 Concluding remarks

The ubiquitin–proteasome pathway plays a fundamental role in the regulation of many viruses [4], [5]. With the recent approval of second-generation proteasome inhibitors in the treatment of certain cancers [44], there is renewed interest for the identification of small molecule compounds that inhibit specific enzymes of the ubiquitin–proteasome pathway to restrict the pathogenesis of infectious diseases within humans. Consequently, understanding the biochemistry of viral regulators that utilize this pathway to stimulate the progress of infection is likely to provide opportunities for the identification and development of novel and efficacious antiviral compounds. Here we describe a protocol utilizing near-IR imaging that provides a highly sensitive and linear (over three-orders of magnitude; Fig. 3E) signal for the accurate quantification of ICP0 RING-finger ubiquitin ligase activity in solution. This methodology will prove valuable in the future identification of regions within ICP0 that influence its biochemical activity, substrate targeting properties, and quantitation of mutants thereof; for example ICP0-RING mutants with reduced E2 or substrate binding affinities [9]. Notably, this protocol can be readily adapted to study other viral and cellular E3 ubiquitin ligases, and applied in the biochemical characterization of small molecule compounds identified to inhibit this important family of enzymes.
